# There is no Neogene denudation conundrum

**DOI:** 10.1073/pnas.2202387119

**Published:** 2022-08-08

**Authors:** Friedhelm von Blanckenburg, Julien Bouchez, Jane K. Willenbring, Daniel E. Ibarra, Jeremy K. Caves Rugenstein

**Affiliations:** ^a^Earth Surface Geochemistry, GFZ German Research Centre for Geosciences, 14473 Potsdam, Germany;; ^b^Institut de Physique du Globe de Paris, CNRS, Université de Paris, 75005 Paris, France;; ^c^Department of Geological Sciences, Stanford University, Stanford, CA 94305;; ^d^Department of Earth, Environmental and Planetary Science, Brown University, Providence, RI 02912;; ^e^Department of Geosciences, Colorado State University, Fort Collins, CO 80523

Li et al. (1) contend that the marine ^10^Be/^9^Be system presents a conundrum, recording constant global weathering and denudation rates over the past 12 My (2), whereas other marine sedimentary isotope records (e.g., Li, Sr, and Os) collectively seem to indicate an increase in late Cenozoic weathering and denudation. They resolve this conundrum with a model focused on demonstrating that oceanic ^10^Be/^9^Be is insensitive to changes in weathering and denudation rates. We believe this conclusion is untenable for three reasons.

First, the model in ref. 1 contains several design errors. In particular, equations 8 and 11 are dimensionally incorrect. Further, α (erosion to denudation; figure S1 of ref. 1) obtained by a logarithmic regression is allowed to exceed 1, yielding negative weathering rates at high denudation. Also, equation 10 assumes the coastal reservoir to be proportional to coastal length, although coastal length is highly fractal in nature. Finally, both their methods to estimate Φ_del_, the efficiency with which Be escapes the coastal trap, result in a larger mismatch between measured and modeled ocean ^10^Be/^9^Be than those reported in our original study (3), even with unchanged parameters and ocean basin ^10^Be/^9^Be data, implying their mass balance may be invalid.

Second, to explore the dependence of ^9^Be delivery on sediment yield using dimensionally correct equations, we revert to the original equations 5 and A13 of ref. 3 and find that it is neither unique nor probable that ocean ^10^Be/^9^Be is insensitive to denudation over three orders of magnitude (Fig. 1). Ref. 1 assumes that riverine and coastal particle concentration is linearly dependent on sediment yield (equations 9 and 11). However, regressing the global river’s particle concentration against sediment yield results in a power law exponent of 0.76 (4). This relationship results in ^10^Be/^9^Be that is sensitive to denudation (Fig. 1). Further, a particle concentration effect (5) controlling the Be partition coefficient cannot be discounted—unlike in ref. 1—if colloids transport ^9^Be into the ocean, resulting in an even greater sensitivity (model e in Fig. 1) to denudation.

**Fig. 1.**
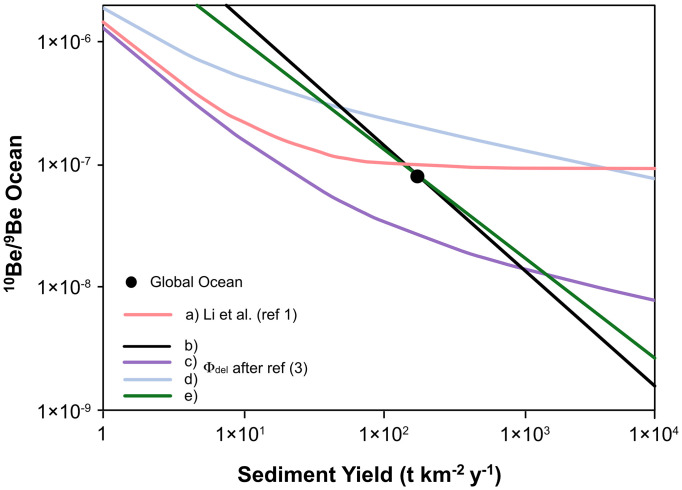
Dependence of ocean ^10^Be/^9^Be on sediment yield. The red curve (a) is from Li et al. (1); their model incorrectly assumes that coastal particle concentration is linearly dependent on sediment yield and that a particle concentration effect (5) is absent. The other curves are calculated using the equations and data from the original model (3) and global ocean values without the South Pacific (table 1 of ref. 3). In curve b, Φ_del_ is constant at 0.063. In all other curves Φ_del_ depends on partition coefficient $K_{d\;\mathrm{coast}}^{Be}$ and on coastal particle concentration (equation A13 of ref. 3) that scales with sediment yield by a power law exponent of 0.76 derived by regressing global river data (4). In curve c, $K_{d\;}^{Be}(\mathrm{coast})$ is 10^4^. In curve d, $K_{d\;}^{Be}(\mathrm{coast})$ is 10^5^. In curve (e), $K_{d\;}^{Be}(\mathrm{coast})$ depends on the particle concentration effect (5). The black point shows global ocean data as calibration to curves b and e without the South Pacific for dearth of data (3), whereas the ref. 1 model includes the South Pacific. All models except the Li et al. model (1) show sensitivity of ^10^Be/^9^Be to sediment yield and hence to weathering flux.

Third, “boundary exchange”—the release of “reactive” terrigenous Be from particles into seawater during early marine diagenesis—presents another input trajectory of ^9^Be (Fig. 2) (3). Ref. 1 ignores this pathway, although it has been shown to operate for several radiogenic and stable isotope systems (6), including beryllium (7).

**Fig. 2.**
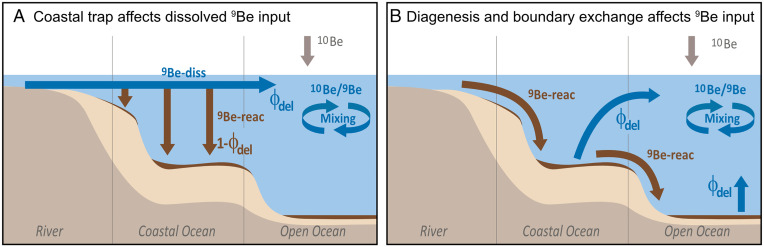
The main entry pathways of ^9^Be into the open ocean after ref. 3. (*A*) The dissolved Be input pathway where Φ_del_ is an estimate of the efficiency by which dissolved Be escapes coastal trapping; this is addressed by the model in ref. 1. (*B*) Input of “reactive” (sediment-bound) ^9^Be and redissolution during “boundary exchange” (6). In this scenario the extent of the Be leakeage from sediment (blue arrows) sets the value of Φ_del_. Although we still lack models that quantify Φ_del_ associated with pathway (*B*), we know that in this case the input of terrigenous Be (low ^10^Be/^9^Be) into the ocean (high ^10^Be/^9^Be) does not depend on the extent of coastal trapping (3).

Even if we do not yet fully understand the ^10^Be/^9^Be system, we know that ^87^Sr/^86^Sr and ^178^Os/^186^Os do not allow one to disentangle changes in flux from changes in lithological source (8) and that ^7^Li/^6^Li does not track continental mass fluxes (9). Only if one accepts the alternative model (1) that Neogene weathering fluxes have increased does the resulting imbalance in the carbon cycle require poorly constrained compensatory fluxes like sulfide weathering, organic carbon oxidation, or reduced basalt weathering. In contrast, the constant weathering flux compatible with ^10^Be/^9^Be (2) ensures balanced carbon fluxes; in turn, global cooling resulted from an increasingly reactive land surface driven by a change in the global ratio between denudation and weathering (10) as indicated by ^7^Li/^6^Li (9). Given the simplicity of this scenario one may ask why the other tracers cited do not present the conundrum, rather than ^10^Be/^9^Be, which in this group of weathering tracers is the only mass flux proxy.

## References

[1] S. Li, S. L. Goldstein, M. E. Raymo, Neogene continental denudation and the beryllium conundrum. Proc. Natl. Acad. Sci. 118, 10.1073/pnas.2026456118 (2021). PMC8545494 34649990

[2] J. K. Willenbring, F. von Blanckenburg, Long-term stability of global erosion rates and weathering during late-Cenozoic cooling. Nature 465, 211–214 (2010). 20463736 10.1038/nature09044

[3] F. von Blanckenburg, J. Bouchez, River fluxes to the sea from the ocean’s 10Be/9Be ratio. Earth Planet. Sci. Lett. 387, 34–43 (2014).

[4] J. D. Milliman, K. L. Farnsworth, River Discharge to the Coastal Ocean: A Global Synthesis (Cambridge University Press, Cambridge, UK, 2011).

[5] N. Hawley, J. A. Robbins, B. J. Eadie, The partitioning of 7 beryllium in fresh water. Geochim. Cosmochim. Acta 50, 1127–1131 (1986).

[6] C. Jeandel, Overview of the mechanisms that could explain the ‘Boundary Exchange’ at the land-ocean contact. Phil. Trans. R. Soc. A. 374, 20150287 (2016). 29035253 10.1098/rsta.2015.0287 PMC5069524

[7] W. Y. Kong, L. P. Zhou; AsterTeam, Tracing water masses and assessing boundary scavenging intensity with beryllium isotopes in the northern South China Sea. J. Geophys. Res. Oceans 126, e2021JC017236 (2021).

[8] C. P. Bataille, A. Willis, X. Yang, X.-M. Liu, Continental igneous rock composition: A major control of past global chemical weathering. Sci. Adv. 3, e1602183 (2017). 28345044 10.1126/sciadv.1602183 PMC5342656

[9] J. K. Caves Rugenstein, D. E. Ibarra, F. von Blanckenburg, Neogene cooling driven by land surface reactivity rather than increased weathering fluxes. Nature 571, 99–102 (2019). 31270485 10.1038/s41586-019-1332-y

[10] L. R. Kump, M. A. Arthur, “Global chemical erosion during the Cenozoic: Weatherability balances the budgets” in Tectonic Uplift and Climate Change, W. F. Ruddiman, Ed. (Plenum Press, 1997), pp. 399–426.

